# MDIPID: Microbiota‐drug interaction and disease phenotype interrelation database

**DOI:** 10.1002/imt2.70019

**Published:** 2025-03-21

**Authors:** Jiayi Yin, Hui Ma, Yuting Qi, Qingwei Zhao, Su Zeng, Fengcheng Li, Feng Zhu

**Affiliations:** ^1^ Department of Clinical Pharmacy, The First Affiliated Hospital Zhejiang University School of Medicine, Zhejiang University Hangzhou China; ^2^ College of Pharmaceutical Sciences, National Key Laboratory of Advanced Drug Delivery and Release Systems Zhejiang University Hangzhou China; ^3^ Children's Hospital, Zhejiang University School of Medicine Zhejiang University Hangzhou China

## Abstract

The intricate bidirectional relationships among microbiota, microbial proteins, drugs, and diseases are essential for advancing precision medicine and minimizing adverse drug reactions. However, there are currently no data resources that comprehensively describe these valuable interactions. Therefore, the Microbiota‐Drug Interaction and Disease Phenotype Interrelation Database (MDIPID) database was developed in this study. MDIPID is distinctive in its ability to elucidate the complex interactions among microbiota, microbial proteins, drugs/substances, and disease phenotypes, thereby providing a comprehensive interconnected network that facilitates the identification of microbial therapy targets and advances personalized medicine. This comprehensive resource is expected to become a popular repository for researchers aiming to identify microbial therapeutic targets, predict drug efficacy, and develop new therapies, thereby facilitating the advancement of personalized medicine. MDIPID can be accessed free without any login requirement at: https://idrblab.org/mdipid/.

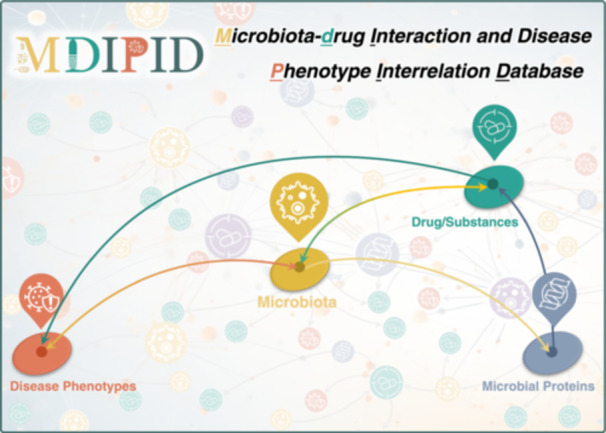

## ETHICS STATEMENT

No animals or humans were involved in this study.

To the Editor,

Accumulating evidence has demonstrated that the human microbiota plays a vital role in disease development and individualized drug responses [1]. Indeed, the relationships among microbiota (and their proteins), drugs, and diseases are intricate and bidirectional. An in‐depth and thorough understanding of these interactions is increasingly regarded as a key factor in advancing precision medicine and reducing adverse drug reactions, attracting considerable attention [2]. There are three major types of interactions: (i) microbiota and their related proteins modulation on drug response (MMDR), which is crucial for minimizing drug‐drug interactions and drug resistance [3]; (ii) drug or other exogenous substances impact on microbiota (DEIM), which is fundamental to understanding the pathophysiology of disease and improving the efficacy of drug therapy [4]; and (iii) microbiota‐disease associations (MBDA), which are essential for identifying predictive biomarkers and helping to stratify patients [5]. Since these complex interactions jointly determine drug response and disease development, accumulating relevant information will significantly enhance clinical drug response predictions and provide new insights into disease therapies.

Moreover, because of the impact of these complex interactions on disease development and drug response, the network formed by these interactions has garnered significant interest and emerged as a promising research direction [6]. For instance, colorectal cancer patients often experience diarrhea during irinotecan chemotherapy [1]. This occurs because irinotecan is metabolized into the toxic compound SN‐38 by the action of β‐glucuronidase enzymes (GUSs) produced by gut microbiota, such as *Escherichia coli* [7]. Consequently, several strategies have been proposed to mitigate these adverse reactions. These strategies leverage comprehensive interaction networks to identify exogenous substances that could safely modulate human microbial taxa and its associated proteins [3]. One approach is to reduce the abundance of pathogenic microbiota by administering exogenous antibiotics, thereby alleviating disease symptoms [8]. Another approach is to supplement with probiotics or use GUSs inhibitors to reduce GUSs activity, thus mitigating the occurrence of diarrhea [9]. Therefore, in addition to the crucial role played by complex bidirectional interactions in drug response and disease development in clinical studies, the networks based on these interactions can further help to facilitate the identification of potential microbiota therapeutic targets and develop new therapies.

Currently, there are several databases available that offer information on the interactions between microbiota, drugs, and diseases, most of which remain freely accessible and are actively maintained. Some of these databases, such as microbe‐drug association database (MDAD) [10] and gutMDisorder [11], offer insights into microbiota‐drug associations but do not capture the complex interaction between microbiota and drugs; some others, such as Peryton [12] and Disbiome [13], provide data on approximately 1000 microbiota and 300 disease associations (a more comprehensive list of the relevant databases is shown in Supporting Information). Microbiota‐active substance interaction (MASI) [14] is the only database that describes the exogenous substances interacting with 806 gut microbiotas. However, to the best of our knowledge, there is currently no database that comprehensively presents the complex bidirectional interactions between drugs, microbiota, and diseases while also including microbial proteins and underlying mechanisms, as well as information on the networks based on these interactions. Given the crucial importance of these interactions and network to disease development and drug response, there is an urgent need for a database that captures these complex interactions and networks to support rational clinical drug delivery and the development of new drugs.

**Figure 1:**
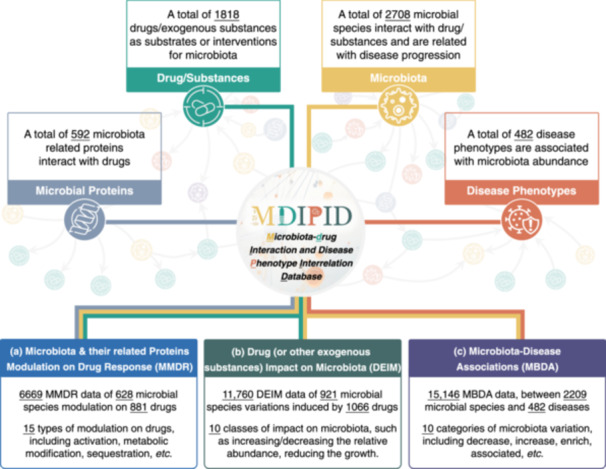
The primary data components of microbiota‐drug Interaction and Disease Phenotype Interrelation Database (MDIPID) and their corresponding statistics. MDIPID consists of four key elements: microbiota (yellow), drugs/substances (green), disease phenotypes (red), and microbial proteins (gray). Additionally, there are complex interaction data composed of these elements: (a) microbiota and their related protein modulation on drug response (MMDR) (blue), (b) drug or other exogenous substances impact on microbiota (DEIM) (dark green), and (c) microbiota‐disease associations (MBDA) (purple). Detailed statistical data for each component are also provided.

In this study, the Microbiota‐Drug Interaction and Disease Phenotype Interrelation Database (MDIPID) was therefore introduced to systematically collect and provide the complex bidirectional interactions among microbiota, drugs, and diseases, as well as microbial proteins and underlying mechanisms (Figure 1). Specifically, (a) 6669 MMDR data records detailing the modulation of 881 drugs by 628 microbial species are presented, involving 592 microbial proteins (MBPS) from 282 microbial species and 15 types of drug modulation, including metabolic modification, sequestration, and activation; (b) 11,760 DEIM data records describing microbiota variations induced by 1066 drugs/exogenous substances are provided, covering 43 groups of substances, such as prebiotics, diet components, and environmental toxicants; and (c) 15,146 MBDA data records illustrating associations between 2209 microbial species and 482 diseases are offered, encompassing 10 types of microbiota variation, such as decrease, increase, and enrichment. Furthermore, the three categories of data collected in MDIPID collectively comprise a comprehensive network that includes 1818 drugs/exogenous substances, 2708 microbial species, 482 diseases, and 592 microbial proteins. Overall, the valuable data described in MDIPID is expected to emerge to become a widely used repository that empowers researchers to gain insights into the relationships between drugs, microbiota, and human diseases, ultimately leading to advancements in medicine and the potential for personalized treatment options.

## RESULTS AND DISCUSSION

### Statistical data of microbiota and their related proteins modulation on drug response

Microbiota modulation on drug response plays an essential role in gaining insight into individual variations in drug response [3]. Specifically, the microbiota has been shown to coordinate the pharmacokinetics and pharmacodynamics of drugs through various mechanisms, such as direct biotransformation, deactivation, or bioaccumulation of drug, indirectly affecting the host's metabolic enzymes, transporters, and immune system. Also, there is increasing evidence that highlights the importance of MBPS in drug response. For instance, some microbial enzymes can deactivate chemotherapeutic drugs, thereby reducing their efficacy, while specific microbial transporters can facilitate the uptake of drugs into the microbiota, diminishing the distribution of these drugs throughout the body and their concentration at target sites [15]. Thus, a comprehensive understanding of how microbiota and their associated proteins modulate drugs is essential for advancing personalized medicine and developing novel therapeutic strategies.

**Figure 2:**
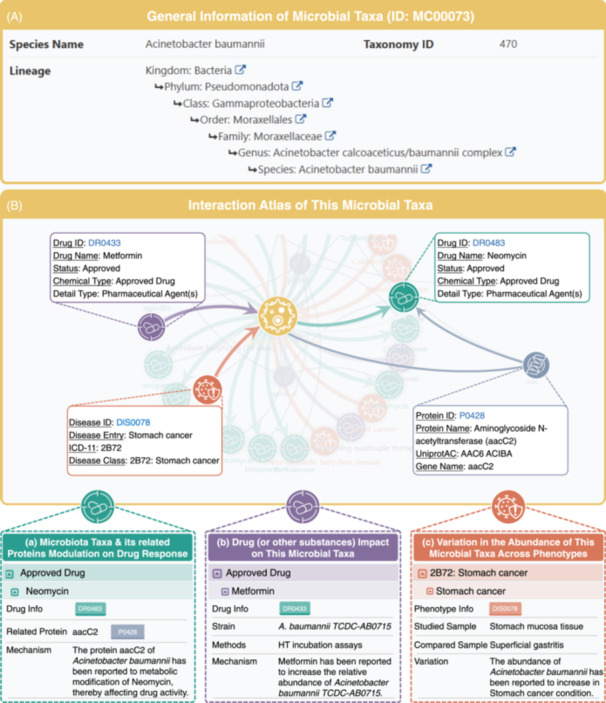
A typical MDIPID page to provide comprehensive details about a specific microbial taxa. (A) general information of microbial taxa (species name, taxonomy ID, and taxonomic lineage); (B) the interactions atlas of this microbial taxa, includes three key interactions: (a) microbial taxa and its related proteins modulation on drug response, (b) drug (or other substances) impact on this microbial taxa, and (c) variation in the abundance of this microbial taxa across phenotypes, along with experimental data and specific regulatory mechanisms involved in each interaction. MDIPID, Microbiota‐Drug Interaction and Disease Phenotype Interrelation Database.

Due to the scattered data regarding the effects of microbiota on drug response in the literature, MDIPID systematically collected and organized relevant data using the methodology described in the “METHODS” section. The data gathered included a total of 628 microbial species belonging to 3 kingdoms and 28 phyla, 881 drugs (approved, clinical trials, or preclinical/investigative), 592 microbial proteins from 63 functional protein families (e.g., the glutamate GABA antiporter family and oxidoreductase), the microbial distribution sites (e.g., colon, small intestine, and brain), the experimental models (e.g., Sprague‐Dawley rats, MCF‐7 breast carcinoma cells, and BALB/c germ‐free mice), the studied phenotypes (e.g., Parkinson disease, diabetes, and colon cancer), the experimental methods (e.g., 16S rDNA sequencing technology and metagenomic sequencing), and the detailed mechanism of microbiota modulation on drug response (Figure 2). To facilitate data exploration, MDIPID offers a variety of strategies for searching on the “Home” page or the “Microbiota” menu (as illustrated in the Data access and retrieval section). These strategies enable researchers to access data on the modulation of drug response by microbiota and their proteins within the database.

### Data of drug or other exogenous substances impact on microbiota

Drug or other exogenous substances on microbiota is a rapidly growing field that holds essential potential for advancing our understanding of disease mechanisms and improving drug therapy [4]. Particularly, drugs/exogenous substances (such as prebiotics, foods, natural products, and environmental toxicants) can interact with microbiota in complex ways and greatly influence its composition and function, which has been reported to be associated with several diseases, such as metabolic syndromes, gastrointestinal cancers, and neurodegenerative conditions [16]. Understanding how the different substances impact the microbiota may provide insight into the mechanism through which they contribute to disease processes. Moreover, as mentioned above, the microbiota plays a key role in drug response, and a thorough knowledge of the drug/substance impact on microbiota will likely facilitate interventions for the human microbiota (e.g., diet, prebiotics, antibiotics, and fecal microbiota transplantation‐based interventions) to improve host response to drug therapy, minimize adverse effects, and reduce drug resistance in immunotherapy, chemotherapy, and radiotherapy. Thus, collecting DEIM data will help researchers develop targeted interventions and will further contribute to the development of precision medicine.

The DEIM data gathered included: 1066 drugs/exogenous substances covering 43 classes (e.g., prebiotics, diet, and environmental toxicants), 921 microbial species belonging to 4 kingdoms, and 48 phyla, the microbiota composition and function variation (e.g., decrease abundance, promote the growth, and inhibit the protein activity of microbiota), the microbial distribution sites (e.g., oral, jejunum, and small intestine), experimental models (e.g., C57BL/6 mice, CD‐1 mice, and zebrafish), the studied phenotypes (e.g., Alzheimer disease, hyperlipidemia, and colorectal cancer), the experimental methods (e.g., metagenomic sequencing and 16S rDNA sequencing technology), and the detailed mechanisms of drugs/substances modulation on the microbiota (Figure S1). Overall, the 11,760 DEIM data of 921 microbial species variation induced by 1066 drugs/substances were described in MDIPID, and several strategies are provided on the “Home” page or the “Drug” menu (as illustrated in the Data access and retrieval section) to help researchers to gain the corresponding information in the database.

### Information on microbiota‐disease associations data

MBDA refers to the fact that when the complex balance of the human microbial system is disrupted, the microbial species becomes unstable, diverse, and more pathogenic, and thus undergoes colony remodeling, which in turn can have a significant impact on the host's disease process [17]. Particularly, a growing number of studies have demonstrated the pathogenic role of specific microbial taxa in a variety of diseases. For example, the growth of *Canidia Albicans* in the intestine has been associated with the development of schizophrenia, COVID‐19, and colorectal cancer [18]; *Malassezia restricta* was identified to exacerbate the inflammation in patients with inflammatory bowel disease [19]; and anti‐*Saccharomyces cerevisiae* antibodies (antibodies against *Saccharomyces cerevisiae*) are increased in patients with Crohn's disease [20] compared to healthy individuals and are therefore used clinically as serological markers for the diagnosis of Crohn's disease. Thus, obtaining a comprehensive understanding of the connection between dysbiosis of microbiota and the pathogenesis of diseases is key for identifying predictive biomarkers and developing innovative therapeutic strategies.

The MBDA data captured in MDIPID encompasses a total of 482 diseases classified under 333 International Classification of Diseases 11th revision (ICD‐11) standardized disease classes, 2209 microbial species belonging to 4 kingdoms and 86 phyla, microbial distribution sites (such as gastrointestinal tract, kidney, and vaginal), 10 categories of microbiota variation (e.g., decrease, increase, and enrichment), experimental methods/samples, the comparative samples, and the detailed mechanisms of microbe‐disease associations (Figure S2). Overall, 15,146 MBDA data between 2209 microbial species and 482 diseases were supplied, and relevant data were retrieved and accessible through the “Home” page or the “Disease” menus (as illustrated in the Data access and retrieval section).

### Collectively construction of the comprehensive and interconnected network

Due to the complexity of the relationships among microbiota (and their proteins), drugs/substances, and diseases, there is a growing necessity and interest in constructing network to encompass and simulate their multifaceted relationships and dynamic interactions. The network is essential for exploring how variations in the microbiota can affect the efficacy and toxicity of drugs, as well as how drugs/substances can modulate the composition and function of the microbiota, which in turn can impact disease development [16]. Additionally, simulating these interactions in a network framework can provide insights into personalized medicine approaches, drug discovery, and precision therapeutics, ultimately leading to improved patient outcomes and the development of novel interventions. Overall, MDIPID comprises a comprehensive and interconnected network, covering 1818 drugs/substances, 2708 microbial species, 482 diseases, and 592 microbial proteins.

### Data access and retrieval

To facilitate efficient data retrieval and access within the MDIPID database, a variety of search methods and data presentation formats are available. Researchers can select the most suitable approach to access the information based on their individual needs and preferences. Detailed descriptions of these options are provided below.

#### For microbiota search and data presentation

Directly from the “Home” page or under the “Microbiota” menu, researchers have the ability to pinpoint pertinent microbial taxa using keyword searches (for instance, microbiota name, drug name, disease name, etc.) or through the deployment of a dropdown menu (categorized by Microbiota Genus and Species Name, Drug Status, and Name, as well as Disease Class and Name) to streamline the retrieval process. On individual microbial taxa pages (Figure 2), researchers are greeted with an array of information, including general information about this microbial taxa and an interaction atlas charting the connections between this microbial taxa, drugs, diseases, and related proteins. This atlas not only contains the key interactions of these elements, such as (a) the modulation of this microbial taxa and its proteins on drug response, (b) the impact of drugs/substances on this microbial taxa, and (c) the variation in the abundance of this microbial taxa across phenotypes, but also incorporates experimental evidence and outlined regulatory mechanisms for these interactions.

#### For drug search and data presentation

Navigable from the “Home” page or via the “Drug” menu, researchers may conduct searches for specific drugs utilizing keywords (like drug name, microbiota name, etc.) or by employing a dropdown menu (organized by Drug Indication and Name, Microbiota Genus, and Species Name). Detailed on the drug‐specific page (Figure S1) are (A) general information of the drug, including names, synonyms, Ro5 violations, current status, structural details, and indications; (B) an interaction atlas detailing drug interactions with microbiota, diseases, and proteins; (C) in‐depth analyses on the drug's impact on microbiota, listing affected species, experimental tools, and the mechanisms behind these effects; and (D) comprehensive information about how microbiota influence drug behavior, detailing specific species involved, experimental approaches, and the underlying mechanisms.

#### For disease search and data presentation

Accessible from the “Home” page or “Disease” menu, researchers can find specific disease using keyword searches (such as disease name, drug name, microbiota name, etc.), or by selecting appropriate options from a dropdown menu (classified by Disease Class and Name, Microbiota Genus, and Species Name, as well as Drug Status and Name). The disease‐specific page (Figure S2) contains the following sections: (A) the general information about the disease (including the disease name, ICD‐11 code, and disease class); (B) an interaction atlas of this disease, visualizing the multidimensional interactions between the disease entity, microbiota, and drug; (C) the detailed descriptions of microbiota abundance variants across this disease, which includes the impacted microbiota, experimental materials, and elaborate mechanisms; (D) a list of drug(s) that treat this disease, indicating the drug name, type, and status.

#### For microbiota‐related protein search and data presentation

From either the “Home” page or the “Protein” menu, researchers can identify microbial proteins through keyword searches (e.g., protein name, drug name, microbiota name, etc.), or via the dropdown menu (sorted by Protein Family and Name, Microbiota Genus, and Species Name, along with Drug Status and Name). Displayed on the protein‐specific page (Figure S3) are (A) general data of protein (name, gene, microbial origins, functional family, tissue distribution, etc.); (B) an interaction atlas that sheds light on the relationships between the protein, microbiota, and drugs; and (C) expansive insights into how the protein influences drugs, detailing the drug's name and status, associated microbiota, metabolic reactions, affinity data, and the mechanisms at play.

## CONCLUSION

Recently, advancement in next‐generation sequencing technology has revolutionized our understanding of the human microbiota and its role in disease progression and drug therapy [1]. This breakthrough has unveiled intricate interactions among the microbiota, various diseases, and therapeutic approaches [16]. There is growing evidence that rationally modulating the composition and function of the microbiota can improve drug efficacy and disease prognosis [1]. However, deciphering these complex issues requires an in‐depth and comprehensive understanding of the specific interactions among the microbiota (and their proteins), drugs, and diseases. To address this need, the MDIPID database was developed. This database provides a multifaceted view of the complex relationships among microbiota (and their proteins), drugs, and diseases, forming an intricate network that provides valuable insights into the therapeutic potential of microbiota manipulation in disease treatment. With the rapid adoption of artificial intelligence in biomedical research, the interconnected data described by MDIPID holds tremendous potential. It enables researchers to exploration of the various interactions among microbiota (and their proteins), drugs, and diseases, accurately predict drug efficacy, and identify potential therapeutic targets within the microbial community. This paves the way for the development of new therapeutic strategies and the advancement of personalized medicine. The MDIPID database is poised to become an essential data resource for researchers seeking to understand the complex relationships between drugs, microbiota, and diseases. By leveraging these insights, it can help to drive medical advancements, ultimately leading to more effective treatments and better patient outcomes.

## METHODS

Comprehensive procedures for data collection and preprocessing, data standardization, as well as website architecture and its implementation, can be found in the Supplementary Information.

## AUTHOR CONTRIBUTIONS

**Jiayi Yin**: Writing—original draft; data curation. **Hui Ma**: Data curation. **Yuting Qi**: Data curation. **Qingwei Zhao**: Data curation; writing—review and editing. **Su Zeng**: Data curation; conceptualization. **Fengcheng Li**: Data curation; visualization; writing—review and editing. **Feng Zhu**: Data curation; conceptualization; writing—review and editing.

## CONFLICT OF INTEREST STATEMENT

The authors declare no conflicts of interest.

## Supporting information


**Figure S1.** A typical illustrative diagram of the abundant drug information in MDIPID.
**Figure S2.** A typical page showing the rich disease phenotype information in MDIPID.
**Figure S3.** A typical page illustrating the microbial protein data in MDIPID.

## Data Availability

The data that support the findings of this study are available from the corresponding author upon reasonable request. MDIPID can be freely accessed at: https://idrblab.org/mdipid/. Supplementary materials (materials, methods, and figures) may be found in the online DOI or iMeta Science http://www.imeta.science/.
