# Supplementary material for: MDIPID: Microbiota‐drug interaction and disease phenotype interrelation database
Source: Imeta. 2025 Mar 21;4(2):e70019. doi: 10.1002/imt2.70019 (PMC11995188; doi:10.1002/imt2.70019)
Supplement: Supplementary file 1 — Figure S1. A typical illustrative diagram of the abundant drug information in MDIPID. Figure S2. A typical page showing the rich disease phenotype information in MDIPID. Figure S3. A typical page illustrating the microbial protein data in MDIPID. [file IMT2-4-e70019-s001.docx]

Supporting Information to

**MDIPID****:** **Microbiota-drug Interaction and Disease Phenotype Interrelation Database**

**Running title**: MDIPID: microbiota-drug-disease interaction database

Jiayi Yin^1^, Hui Ma^1^, Yuting Qi^1,2^, Qingwei Zhao^1^, Su Zeng^2^*, Fengcheng Li^3^*, and Feng Zhu^2^*

^1^Department of Clinical Pharmacy, The First Affiliated Hospital, Zhejiang University School of Medicine, Zhejiang University, Hangzhou 310003, China

^2^College of Pharmaceutical Sciences, National Key Laboratory of Advanced Drug Delivery and Release Systems, Zhejiang University, Hangzhou 310058, China

^3^Children’s Hospital, Zhejiang University School of Medicine, Zhejiang University, Hangzhou 310052, China

*Correspondence: [zhufeng@zju.edu.cn](mailto:zhufeng@zju.edu.cn) (Feng Zhu), [lifc@zju.edu.cn (Fengcheng Li),](mailto:lifc@zju.edu.cn,) [zengsu@zju.edu.cn](mailto:zengsu@zju.edu.cn) (Su Zeng)

**SUPPLEMENTARY MATERIALS AND METHODS**

**The existing relevant databases**

Currently, there are several databases available that offer information on the interactions between microbiota, drugs, and diseases, most of which remain freely accessible and are actively maintained. Some of these databases, such as microbe-drug association database (MDAD) [1], MADET [2], DrugVirus.info [3], gutMDisorder [4], and PharmacoMicrobiomics [5], offer insights into microbiota-drug associations but do not capture the complex interaction between microbiota and drugs; some others, such as Peryton [6], HMDAD [7], Amadis [8], Disbiome [9], and GMrepo [10], provide data on approximately 1000 microbiota and 300 disease associations. MASI [11] is the only database that describes the exogenous substances interacting with 806 gut microbiotas. However, to the best of our knowledge, there is currently no database that comprehensively presents the complex bidirectional interactions between drugs, microbiota, and diseases, while also including microbial proteins and underlying mechanisms, as well as information on the networks based on these interactions. Given the crucial importance of these interactions and network to disease development and drug response [12,13], there is an urgent need for a database that captures these complex interactions and networks to support rational clinical drug delivery and the development of new drugs.

**Data collection and preprocessing**

Before commencing data collection, we first conducted an exhaustive investigation of existing databases in the field related to the interactions among microbiota, drugs, and diseases. The existing databases provide some associations between microbes and drugs, as well as data on approximately 1000 microbiota and 300 disease associations, which form a solid data foundation for the construction of the MDIPID database. However, due to the diverse sources of these data and the lack of unified standards in data structures, we initially carried out systematic collection and organization of the relevant data. Subsequently, through data normalization (including unifying data formats, standardizing naming conventions, and eliminating redundant information) and deduplication, we ensured that only those relationships between drugs and microbes with reliable literature sources and experimental validation, as well as specific microbial data clearly associated with diseases, were incorporated into the MDIPID database.

Moreover, most existing databases primarily focus on microbiota-drug associations or microbiota-disease associations, while lacking detailed descriptions regarding the specific actions of microbiota with drugs/diseases, the specific impacts of drugs/diseases on the microbiota, the involved microbial proteins and potential mechanisms, as well as network information based on these interactions. This results in the complex bidirectional interactions among drugs, microbiota, and diseases not being comprehensively presented, and the crucial role of microbial proteins in microbiota-drug interactions being severely overlooked. Therefore, based on the aforementioned data, we adopted a combination of automatic retrieval and manual information extraction methods to systematically collect MMDR data, DEIM information, and MBDA content scattered across a vast amount of scientific literature.

In terms of automated retrieval, we have carefully selected and configured our search engines and databases. We utilize the high-quality publications PubMed, which contain a substantial number of rigorously peer-reviewed research articles. Our search algorithms are designed to accurately capture relevant articles from PubMed, and we regularly update our search strategies to align with emerging research trends, ensuring comprehensive data retrieval. For the manual information extraction process, we have assembled a highly qualified team of experts in microbiology, pharmacology, and related fields. These professionals adhere to strict standards when extracting information from the retrieved articles. They classify the extracted data logically, considering various aspects of the research content and data types.

To gather information on the effects of microbiota and their related proteins on drugs, we conducted a comprehensive literature review on PubMed, as much of this information is scattered throughout the literature. MDIPID focus on all FDA-approved drugs (over 2000, with data from the FDA website [14]) and more than 10,000 drugs currently in clinical or preclinical trials (data obtained from ClinicalTrials.gov [15], DrugBank [16], and DrugMap [17]). For the collection of data on the effects of microbiota and their related proteins on drugs, the keyword combinations used in automatic retrieval included ‘microbiota’ + ‘drug metabolism’, ‘microbiota’ + ‘drug transport’, ‘microbiota’+ ‘drug sequestration’, ‘microbiota’ + ‘drug activation’, and combinations of ‘microbiota’ or ‘microbiome’ with the names and synonyms of each drug. Regarding the impact of drugs or other exogenous substances on microbiota, relevant data were systematically retrieved from the PubMed database using a combination of keywords, including ‘drug name’, ‘substances’, ‘prebiotic’, ‘antibiotics’, ‘plant extract’, ‘probiotic’, ‘environmental toxicant’, ‘diet’, ‘food’, and the names of each microbial taxa (or only ‘microbiota/microbe’). Subsequently, to obtain data on the relationships between microbiota and disease, a comprehensive literature search was conducted using various keyword combinations, such as ‘disease name’ + ‘microbiota name/synonyms’, ‘abundance’ + ‘microbiota name/synonyms’, and ‘disease name’ + ‘microbiota name/synonyms’ + ‘associations’. The literature uncovered was manually assessed to extract any information regarding the associations between microbiota and diseases. Ultimately, we retrieved approximately 50,000 relevant articles through automated keyword searches. After excluding non-potential articles (those with lower relevance) and conducting a systematic review, we narrowed it down to around 10,000 articles that met our criteria, ultimately extracting over 28,000 relevant entries from approximately 3500 articles.

**Data standardization**

To facilitate convenient access and analysis of MDIPID data for all users, the collected raw data underwent meticulous cleaning and systematic standardization. This process included microbial species classification, substances classification, drug 2D/3D structures, disease standardization, and so on. Furthermore, each microbial taxa, along with its related drugs/substances or diseases, is accessible through cross-links to several reputable databases. These include NCBI Taxonomy [18], Drugbank [16], DrugMAP [17], CAS Registry Number [19], Drugs@FDA [14], PubChem [20], BRENDA Enzyme database [21], TCDB [22], INTEDE [23], VARIDT [24], ICD-11 [25], and UniProtKB [26]. By thoroughly standardizing the datasets, we ensure that users can accurately and efficiently access and analyze comprehensive data related to microbiota, drugs/substances, and associated diseases.

**Website architecture and implementation**

The website architecture of MDIPID has been meticulously developed on a cloud-based Linux server, specifically utilizing Ubuntu 16.04.3 as its foundational operating system. The platform employs the stable and widely used Apache web server (version 2.4.37) to ensure reliable performance. All data stored and managed within MDIPID is handled by MariaDB (version 15.1), providing robust database management capabilities. The MDIPID web interface is built on the Drupal framework (version 8.8.5) for the backend and utilizes the Bootstrap framework (version 4.4.1) for the front-end, ensuring a responsive and user-friendly experience. Additionally, the interactive interaction network graph and radar chart in MDIPID were implemented using the open-source JavaScript visualization library ECharts (version 5.3.2). For public accessibility, the website is deployed at https://idrblab.org/mdipid/ and does not require a login for access.

**REFERENCES**

1. Sun, Ya-Zhou, De-Hong Zhang, Shu-Bin Cai, Zhong Ming, Jian-Qiang Li, and Xing Chen. 2018. “MDAD: a special resource for microbe-drug associations.” *Frontiers in Cellular and Infection Microbiology* 8: 424. <https://doi.org/10.3389/fcimb.2018.00424>

2. Zhang, Jie, Xiqian Chen, Jiaxin Zou, Chen Li, Wanying Kang, Yang Guo, Sheng Liu, et al. 2022. “MADET: a manually curated knowledge base for microbiomic effects on efficacy and toxicity of anticancer treatments.” *Microbiology Spectrum* 10: e0211622. <https://doi.org/10.1128/spectrum.02116-22>

3. Ianevski, Aleksandr, Ronja M Simonsen, Vegard Myhre, Tanel Tenson, Valentyn Oksenych, Magnar Bjørås, and Denis E Kainov. 2022. “DrugVirus.info 2.0: an integrative data portal for broad-spectrum antivirals (BSA) and BSA-containing drug combinations (BCCs).” *Nucleic Acids Research* 50: W272-W275. <https://doi.org/10.1093/nar/gkac348>

4. Qi, Changlu, Yiting Cai, Kai Qian, Xuefeng Li, Jialiang Ren, Ping Wang, Tongze Fu, et al. 2023. “gutMDisorder v2.0: a comprehensive database for dysbiosis of gut microbiota in phenotypes and interventions.” *Nucleic Acids Research* 51: D717-D722. <https://doi.org/10.1093/nar/gkac871>

5. Rizkallah, Mariam R, Soha Gamal-Eldin, Rama Saad, and Ramy K Aziz. 2012. “The PharmacoMicrobiomics portal: a database for drug-microbiome interactions.” *Current Pharmacogenomics & Personalized Medicine* 10: 195-203. <https://doi.org/10.2217/pgs-2019-0027>

6. Skoufos, Giorgos, Filippos S Kardaras, Athanasios Alexiou, Ioannis Kavakiotis, Anastasia Lambropoulou, Vasiliki Kotsira, Spyros Tastsoglou, et al. 2021. “Peryton: a manual collection of experimentally supported microbe-disease associations.” *Nucleic Acids Research* 49: D1328-D1333. <https://doi.org/10.1093/nar/gkaa902>

7. Ma, Wei, Lu Zhang, Pan Zeng, Chuanbo Huang, Jianwei Li, Bin Geng, Jichun Yang, Wei Kong, Xuezhong Zhou, Qinghua Cui. 2017. “An analysis of human microbe-disease associations.” *Briefings in Bioinformatics* 18: 85-97. <https://doi.org/10.1093/bib/bbw005>

8. Li, Long, Qingxu Jing, Sen Yan, Xuxu Liu, Yuanyuan Sun, Defu Zhu, Dawei Wang, et al. 2021. “Amadis: a comprehensive database for association between microbiota and disease.” *Frontiers in Physiology* 12: 697059. <https://doi.org/10.3389/fphys.2021.697059>

9. Janssens, Yorick, Joachim Nielandt, Antoon Bronselaer, Nathan Debunne, Frederick Verbeke, Evelien Wynendaele, Filip Van Immerseel, et al. 2018. “Disbiome database: linking the microbiome to disease.” *BMC Microbiology* 18: 50. <https://doi.org/10.1186/s12866-018-1197-5>

10. Dai, Die, Jiaying Zhu, Chuqing Sun, Min Li, Jinxin Liu, Sicheng Wu, Kang Ning, et al. 2022. “GMrepo v2: a curated human gut microbiome database with special focus on disease markers and cross-dataset comparison.” *Nucleic Acids Research* 50: D777-D784. <https://doi.org/10.1093/nar/gkab1019>

11. Zeng, Xian, Xue Yang, Jiajun Fan, Ying Tan, Lingyi Ju, Wanxiang Shen, Yali Wang, et al. 2021. “MASI: microbiota-active substance interactions database.” *Nucleic Acids Research* 49: D776-D782. <https://doi.org/10.1093/nar/gkaa924>

12. Zhao, Lin-Yong, Jia-Xin Mei, Gang Yu, Lei Lei, Wei-Han Zhang, Kai Liu, Xiao-Long Chen, et al. 2023. “Role of the gut microbiota in anticancer therapy: from molecular mechanisms to clinical applications.” *Signal Transduction and Targeted Therapy* 8: 201. <https://doi.org/10.1038/s41392-023-01406-7>

13. Ratiner, Karina, Dragos Ciocan, Suhaib K Abdeen, and Eran Elinav. 2024. “Utilization of the microbiome in personalized medicine.” *Nature Reviews Microbiology* 22: 291-308. <https://doi.org/10.1038/s41579-023-00998-9>

14. Schwartz, Lisa M, Steven Woloshin, Eugene Zheng, Tony Tse, and Deborah A Zarin. 2016. “ClinicalTrials.gov and Drugs@FDA: a comparison of results reporting for new drug approval trials.” *Annals of Internal Medicine* 165: 421-430. <https://doi.org/10.7326/M15-2658>

15. Tse, Tony, Kevin M Fain, and Deborah A Zarin. 2018. “How to avoid common problems when using ClinicalTrials.gov in research: 10 issues to consider.” *British Medical Journal* 361: k1452. <https://doi.org/10.1136/bmj.k1452>

16. Knox, Craig, Mike Wilson, Christen M Klinger, Mark Franklin, Eponine Oler, Alex Wilson, Allison Pon, et al. 2024. “DrugBank 6.0: the DrugBank knowledgebase for 2024.” *Nucleic Acids Research* 52: D1265-D1275. <https://doi.org/10.1093/nar/gkad976>

17. Li, Fengcheng, Jiayi Yin, Mingkun Lu, Minjie Mou, Zhaorong Li, Zhenyu Zeng, Ying Tan, et al. 2023. “DrugMAP: molecular atlas and pharma-information of all drugs.” *Nucleic Acids Research* 51: D1288-D1299. <https://doi.org/10.1093/nar/gkac813>

18. Federhen, Scott. 2015. “Type material in the NCBI Taxonomy database.” *Nucleic Acids Research* 43: D1086-1098. <https://doi.org/10.1093/nar/gku1127>

19. Stobaugh, Robert E. 1988. “Chemical abstracts service chemical registry system. 11. substance-related statistics: update and additions.” *Journal of Chemical Information and Modeling* 28: 180-187. <https://doi.org/10.1021/ci00060a003>

20. Kim, Sunghwan, Jie Chen, Tiejun Cheng, Asta Gindulyte, Jia He, Siqian He, Qingliang Li, et al. 2023. “PubChem 2023 update.” *Nucleic Acids Research* 51: D1373-D1380. <https://doi.org/10.1093/nar/gkac956>

21. Chang, Antje, Lisa Jeske, Sandra Ulbrich, Julia Hofmann, Julia Koblitz, Ida Schomburg, Meina Neumann-Schaal, et al. 2021. “BRENDA, the ELIXIR core data resource in 2021: new developments and updates.” *Nucleic Acids Research* 49: D498-D508. <https://doi.org/10.1093/nar/gkaa1025>

22. Saier, Milton H, Vamsee S Reddy, Gabriel Moreno-Hagelsieb, Kevin J Hendargo, Yichi Zhang, Vasu Iddamsetty, Katie Jing Kay Lam, et al. 2021. “The Transporter Classification Database (TCDB): 2021 update.” *Nucleic Acids Research* 49: D461-D467. <https://doi.org/10.1093/nar/gkaa1004>

23. Yin, Jiayi, Fengcheng Li, Ying Zhou, Minjie Mou, Yinjing Lu, Kangli Chen, Jia Xue, et al. 2021. “INTEDE: interactome of drug-metabolizing enzymes.” *Nucleic Acids Research* 49: D1233-D1243. <https://doi.org/10.1093/nar/gkaa755>

24. Yin, Jiayi, Zhen Chen, Nanxin You, Fengcheng Li, Hanyu Zhang, Jia Xue, Hui Ma, et al. 2024. “VARIDT 3.0: the phenotypic and regulatory variability of drug transporter.” *Nucleic Acids Research* 52: D1490-D1502. <https://doi.org/10.1093/nar/gkad818>

25. The, Lancet. 2019. “ICD-11.” *Lancet* 393: 2275. <https://doi.org/10.1016/S0140-6736(19)31205-X>

26. Consortium, UniProt. 2023. “UniProt: the universal protein knowledgebase in 2023.” *Nucleic Acids Research* 51: D523-D531. <https://doi.org/10.1093/nar/gkac1052>


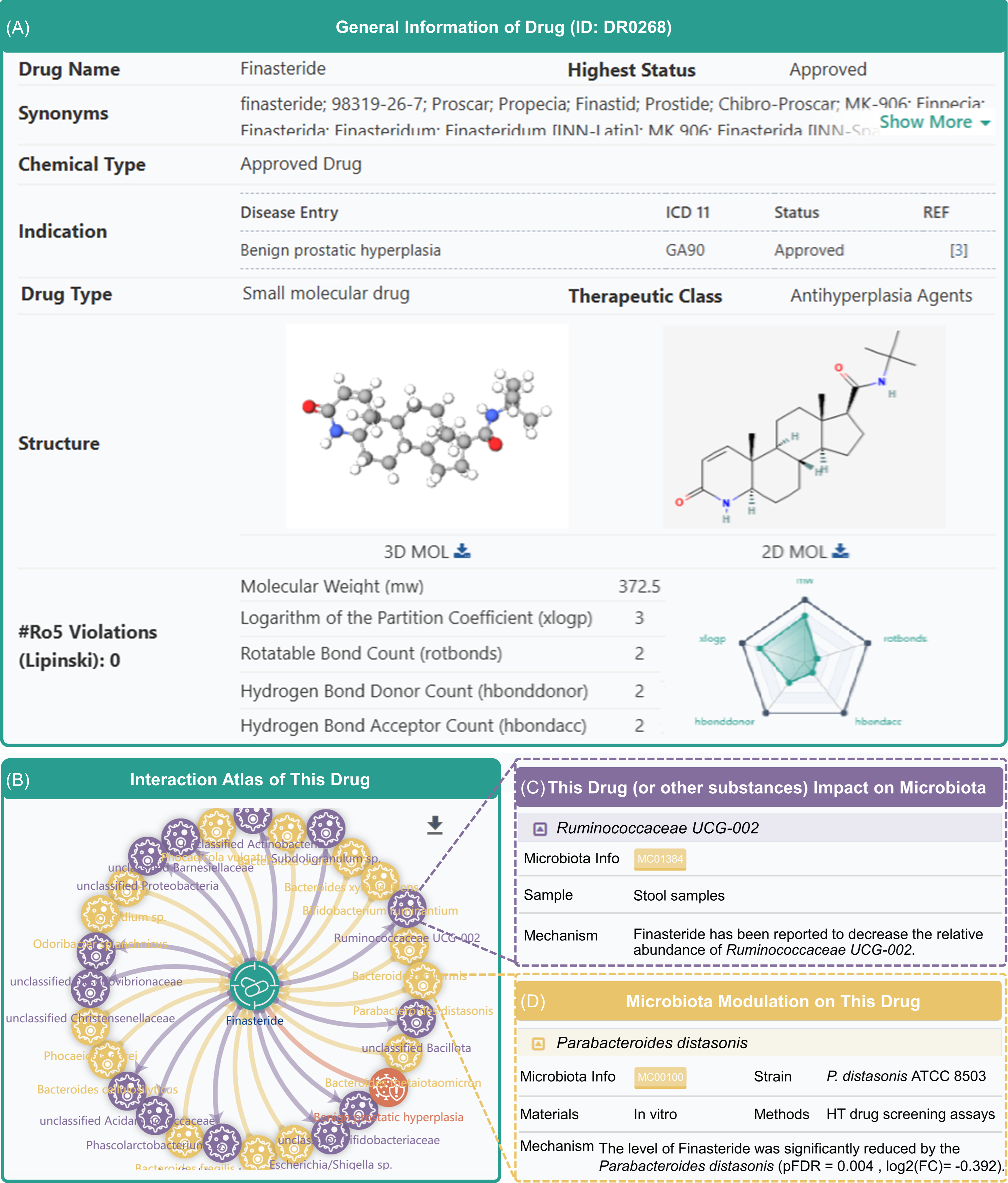


**Figure S1** **A typical** **illustrative diagram of the abundant drug information in MDIPID.** (A) general information of drugs, including drug name, synonyms, drug status, structure, Ro5 violations, and indications; (B) interaction atlas of the drug, which includes an overview of the interactions between drugs, microbiota, diseases, and proteins; (C) detailed insights into the impact of this drug on microbiota, comprising the impacted microbiota, experimental materials, and elaborate mechanisms; (D) elaborate data on microbiota modulation on this drug, including specific microbiota, experimental methods, and materials, as well as detailed mechanisms*.*


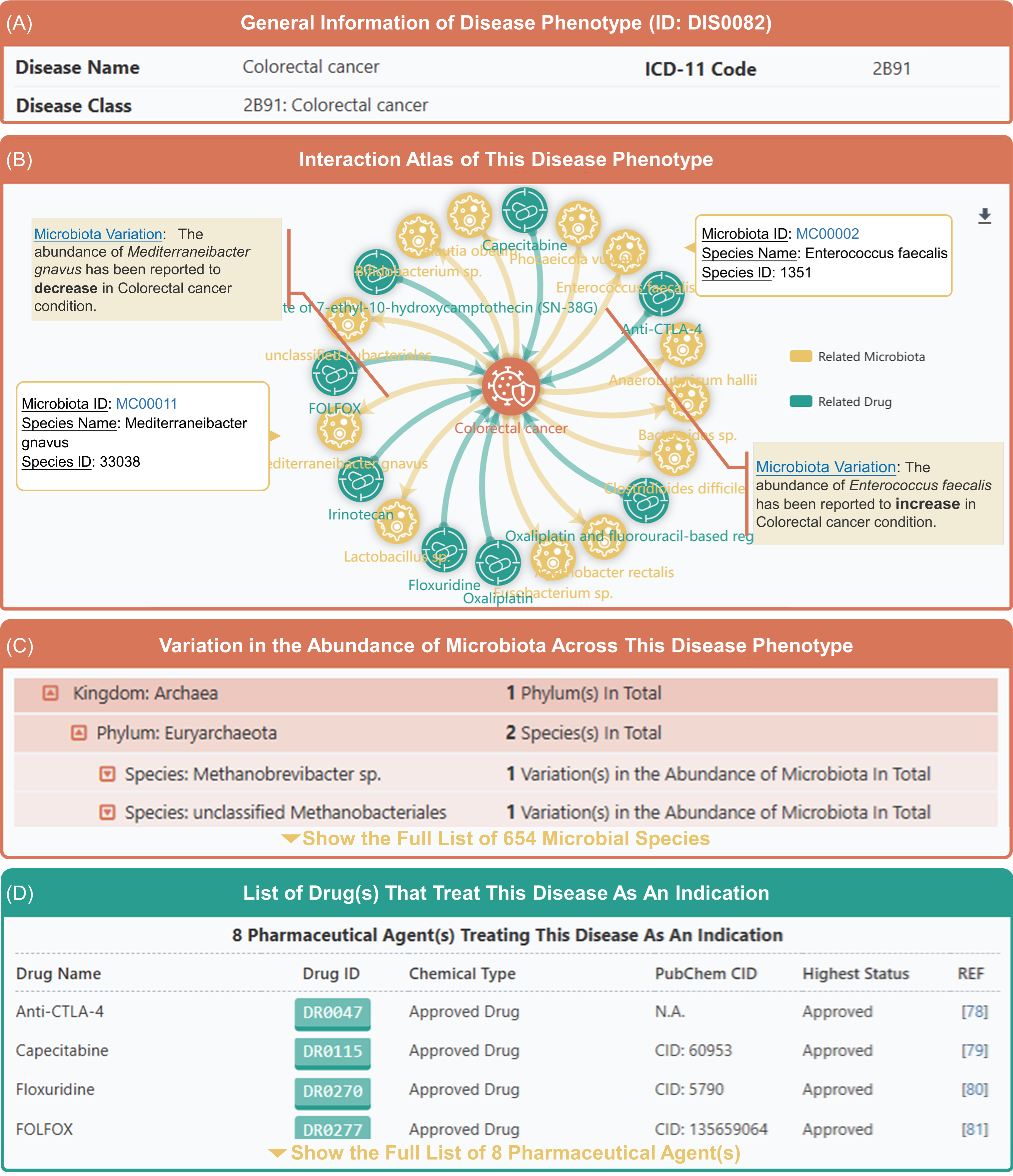


**Figure S2** **A typical page showing the rich disease phenotype information in MDIPID.** (A) general information of disease, including disease name, ICD-11 code, and disease class; (B) interaction atlas of this disease, with an overview of the interactions between this disease, microbiota, and drugs, as well as detailed data on microbiota and microbiota variation; (C) detailed descriptions of microbiota abundance variants across this disease, which included the impacted microbiota, experimental materials, and elaborate mechanisms; (D) the list of drug(s) that treat this disease, including the drug name, type, and status.


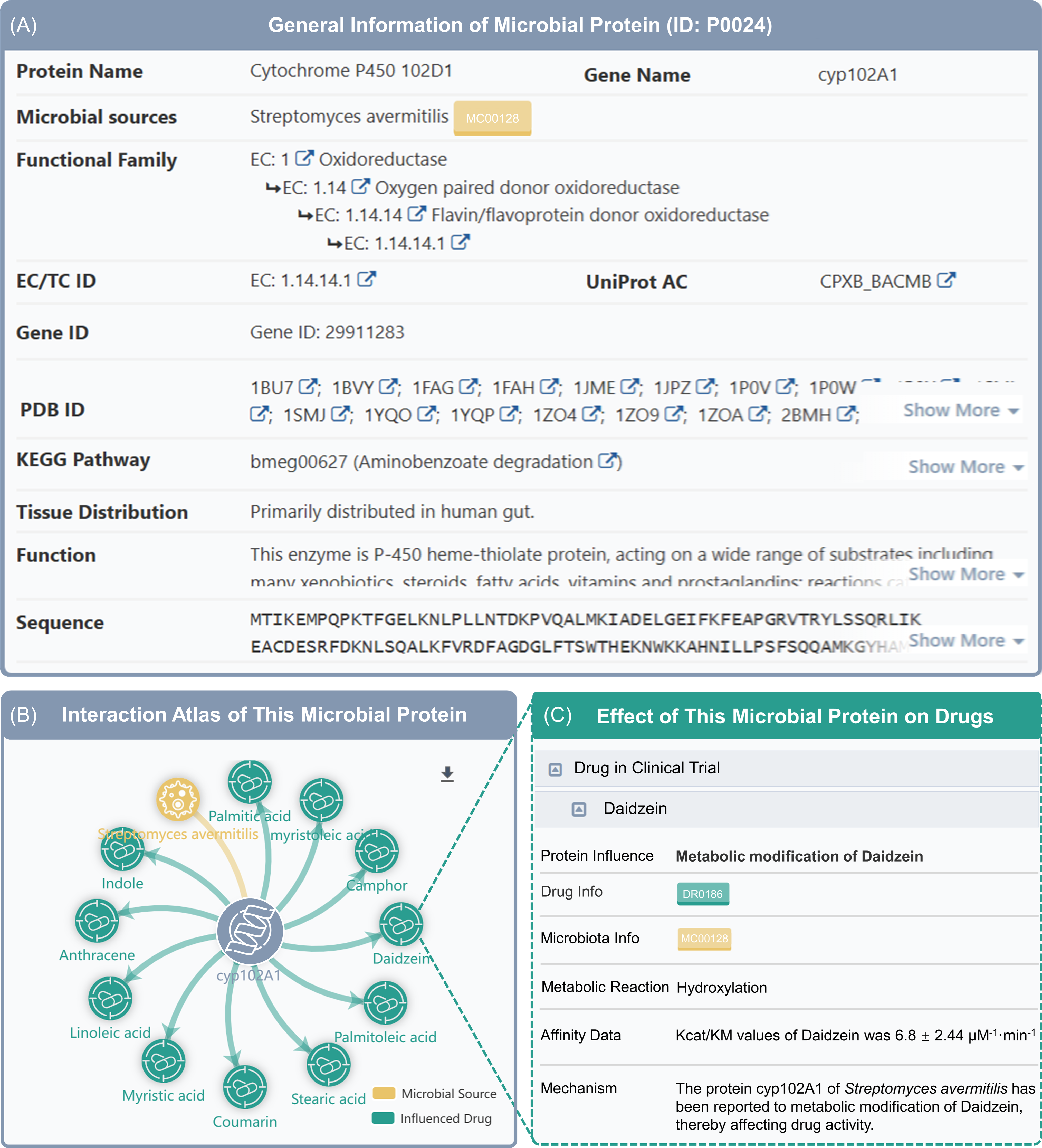


**Figure S3** **A typical page illustrating the microbial protein data in MDIPID.** (A) general information about the protein, including protein name, gene name, microbial sources, functional family, and tissue distribution; (B) interaction atlas of the protein, including an overview of interactions between the protein, microbiota, and drugs; (C) detailed insights into the effect of this protein on the drug, including drug name, drug status, related microbiota, metabolic reaction, affinity data, as well as detailed mechanisms*.*
